# Three Dimensionally Printed Versus Conventional Casts in Pediatric Wrist Fractures

**DOI:** 10.7759/cureus.19090

**Published:** 2021-10-28

**Authors:** Hope E Skibicki, Brian M Katt, Kevin Lutsky, Mark L Wang, Richard McEntee, Alexander R Vaccaro, Pedro Beredjiklian, Michael Rivlin

**Affiliations:** 1 Orthopedic Surgery, Rowan University School of Osteopathic Medicine, Stratford, USA; 2 Department of Orthopedic Surgery, Rutgers Robert Wood Johnson Medical School, New Brunswick, USA; 3 Division of Hand Surgery, Rothman Orthopaedic Institute, Philadelphia, USA; 4 Spine Surgery, Rothman Orthopaedic Institute, Philadelphia, USA

**Keywords:** forearm fracture, wrist fracture, 3d printed cast, upper extremity casting, 3d orthosis

## Abstract

Background and objective

With significant advancement in the field of biomaterials, alternatives to conventional fiberglass casts such as customized three-dimensional (3D) orthotics have been developed. However, there is a scarcity of reported experience regarding 3D-printed orthoses. The purpose of this study was to compare radiographic outcomes and patient satisfaction with fractures treated with either conventional or 3D-printed casts.

Materials and methods

We included 23 limbs from 22 patients, who were aged between 8-18 years, and with a diagnosis of an acute nondisplaced wrist or forearm fracture. Patients were randomized into two groups: consisting of those treated with a 3D-printed orthosis and those with conventional fiberglass cast. Outcomes included X-ray alignment and healing, cast fit, the appearance of the skin, ease of care, and overall satisfaction.

Results

Of note, 10/11 (91%) in the 3D cast group healed in an excellent position, and 1/11 healed in an acceptable position. Also, 11/12 (92%) in the conventional cast group healed in an excellent position, and 1/12 healed in an acceptable position. Radiographically, 11/11 (100%) of the fractures in the 3D group and 11/12 (92%) in the conventional cast group were found to be fully healed. No differences were found in terms of skin irritation by a blinded hand therapist. Patients reported significant differences in skin irritation, comfort, satisfaction, and cast care favoring 3D casts (p<0.05).

Conclusions

3D orthoses offer a promising opportunity to improve patients’ experiences with upper extremity casting while also providing appropriate immobilization.

## Introduction

Pediatric fractures of the forearm and wrist are common injuries, comprising 40% of all fractures in children. The most prevalent injuries are those to the distal metaphysis and physis [1,2]. Nonoperative management through splinting and casting is an integral part of injury treatment [3,4]. Salter-Harris physeal injuries are treated with immobilization even in the absence of radiographic evidence of injury. Traditional casting with plaster and, recently, fiberglass has been highly regarded due to its low cost, strength, and ease of application. However, the cost of manufacturing three-dimensional (3D) casts is now comparable to that of other orthoses. In addition, the cost of 3D casts is expected to further decrease over time.

With the advancement of biomaterials and newer manufacturing technologies, alternatives to conventional casts have been developed. Specifically, the medical applications of 3D printing continue to expand, with the creation of customized prosthetics and orthotics as attractive options [5,6]. Custom 3D-printed orthoses for nondisplaced or incomplete upper extremity fractures may provide comfort along with the advantage of direct visualization and monitoring of the skin while minimizing the need to keep the cast dry. While the technology is presently available, there is scarce anecdotal data about the use of 3D-printed orthoses in the clinical setting for upper extremity pediatric fractures. The purpose of this study was to compare radiographic outcomes and patient satisfaction with fractures treated with either conventional or 3D-printed casts. This was primarily designed as a safety and proof of concept study. We hypothesized that 3D-printed casts have superior properties compared to conventional fiberglass casts.

## Materials and methods

This randomized controlled study commenced after obtaining approval from the Institutional Review Board (IRB) of our institution (Thomas Jefferson University, Philadelphia, PA). The three treating orthopedic surgeons were board-certified and hand fellowship-trained. A fourth board-certified, hand fellowship-trained surgeon, not involved in the patient care, was assigned to review the radiographs. Patients were recruited if they were between the ages of 8-18 years and had the diagnosis of an acute (less than one week old), nondisplaced, or minimally displaced wrist or forearm fracture. Patients were excluded if the fracture required a reduction or surgical treatment, if they had multiple fractures, underlying bone pathology, an underlying metabolic disorder, or declined to participate in the study. Displaced fractures were excluded because these generally require reduction, and these injuries are not appropriate for scanning and 3D-printed casts. No financial benefit was offered nor any burden placed on the patients for participating in the study. Four patients declined to participate, yielding a 22-patient study cohort.

**Figure 1:**
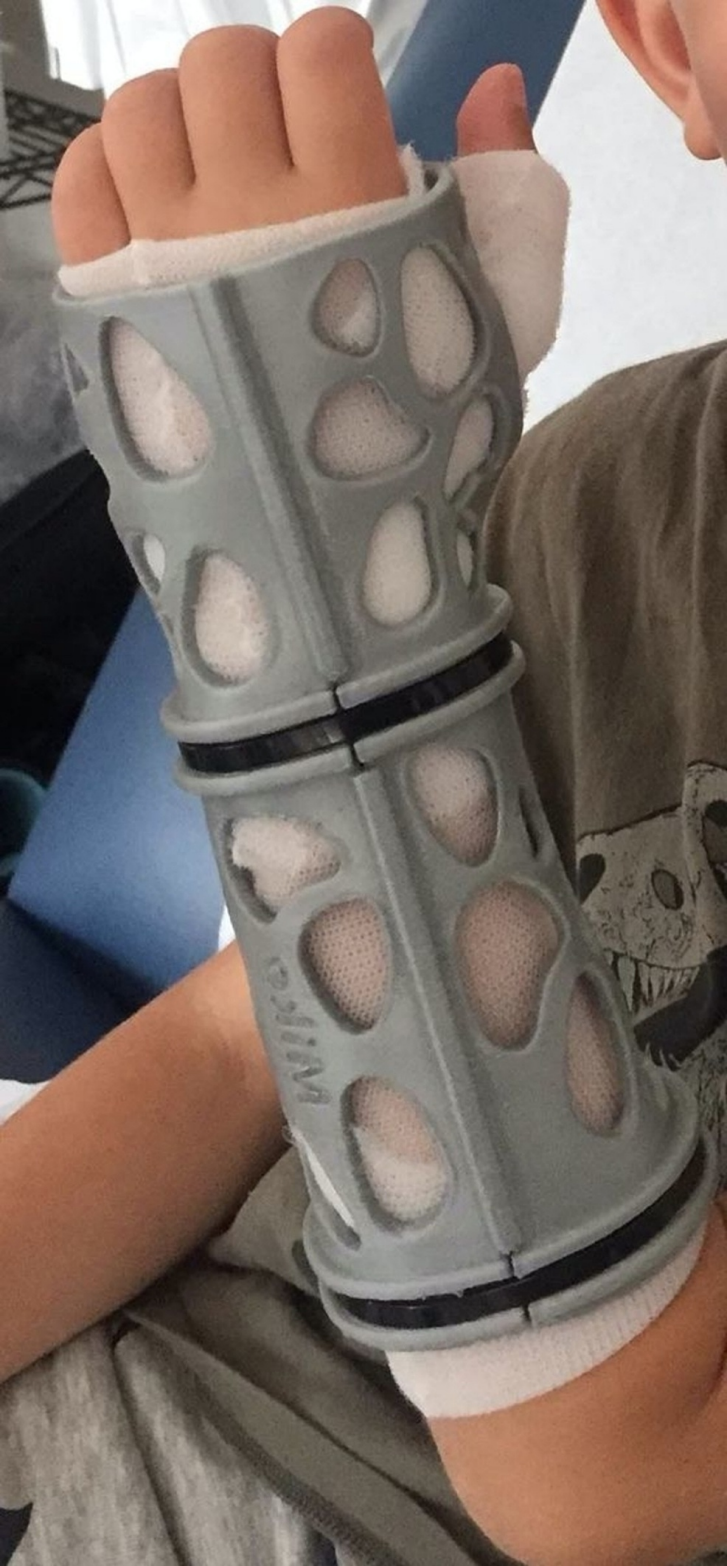
3D-printed orthosis

Parents of the patients were given a detailed information sheet explaining the purpose of the study and the course of treatment and were informed that their children's fractures would be placed either in a 3D-printed orthosis or in a fiberglass cast (Figure 1). Patients were entered into the study after the parent or guardian had signed a consent form as approved by the IRB. Potential harms from treatment were explained to patients prior to the enrollment, including rashes, skin irritation, pressure marks, discomfort, or allergic reactions. Patients were randomized into two different groups by picking a pre-randomized, numbered envelope. The study group consisted of patients treated with a 3D-printed orthosis. The control group consisted of the patients treated with a conventional fiberglass cast. The hand surgeon who reviewed the X-rays and the hand therapy staff involved in the assessments were blinded as to which group each patient belonged. The treating surgeons and orthotists who applied the casts were not blinded, but they were instructed to file a report if the orthosis fit was not satisfactory.

The treatment course entailed an initial encounter in which patients had their first X-ray evaluated by the treating hand surgeon, enrolled into the study, and placed in a temporary splint. The second visit occurred within a week of the initial visit for splint removal and 3D or conventional cast application. The next encounter was four weeks after enrollment and included an evaluation by the treating surgeon, an assessment by a blinded hand therapist, and cast removal. At this encounter, four different assessments were performed. The first was performed by the blinded hand surgeon, which only involved the radiographic analysis, to evaluate fracture type, X-ray alignment, and fracture healing. The second evaluation was completed by the blinded hand therapist to document the appearance of the skin and cast fit according to the integrity of the skin. Of note, the orthosis was removed by the orthotist prior to the therapist or hand surgeon seeing the patient. X-rays were obtained after the casts were removed. The third evaluation was completed with the patients and parents noting the appearance of the skin, comfort of the cast using a visual analog scale (VAS), ease of cast care, overall satisfaction of the cast (VAS), and also assessing if the cast needed to be changed. The orthotist was also asked to note whether there was an inappropriate/unacceptable fit or if the cast had to be changed. A visit was scheduled eight weeks after enrollment, but only if it was deemed necessary by the treating surgeon during the third visit.

To create the 3D cast, a 3D scanner (Einscan Pro, Shining 3D, Hangzhou, China) was used to digitize the arm in a proper position during the first visit. The 3D scan was then modeled and printed using a 3D printer (Ultimaker Ext 3, Utrecht, Netherlands). The 3D-printed orthoses were made from Food and Drug Administration (FDA)-approved polymer materials. The 3D casts had a vented mesh-type design. After molding, the final orthosis was applied by the orthotist in the clinic. They were applied by putting the two halves together and securing them with a tamper-proof locking mechanism. For the conventional cast, traditional casting materials were used, including cast padding overwrapped with a fiberglass roll.

Data were collected in a de-identified database. Statistical analysis was performed using the Fisher's exact test for non-parametric data and the Mann-Whitney U test for non-normally distributed data. A p-value of <0.05 was considered statistically significant.

## Results

The study group consisted of 23 limbs from 22 patients, with 10 patients (11 limbs) in the 3D cast group and 12 in the conventional cast group. Patient demographics are provided in Table 1. Ten of the 11 fractures in the 3D cast group involved the distal radius: three Salter-Harris type I fractures and seven buckle metaphyseal fractures. The remaining fracture was a nondisplaced meta/diaphyseal ulnar fracture. All of the fractures in the conventional cast group included the radius, and two of these also involved the ulna. There were two Salter-Harris type I, two Salter-Harris type II, and the rest were metaphyseal fractures. One of the ulnar fractures involved the styloid, while the second involved the ulnar metaphysis. All of the fractures in both groups were non to minimally displaced and stable. Two patients in the experimental group and three patients in the control group were followed up for eight weeks and the rest for four weeks.

**Table 1:** Patient demographics

Variables	3D cast	Conventional cast
Total patients	10	12
Total limbs	11	12
Gender		
Male	8	7
Female	2	5
Hand dominance		
Right	10	11
Left	0	1
Affected hand		
Right	6	4
Left	5	8
Dominant	6	5
Years		
Mean	11.3	11.5
Standard deviation	3.7	1.9
Minimum	8	9
Maximum	18	14

All fractures healed and remained in either excellent or acceptable alignment as defined by the blinded surgeon and clinically by the non-blinded, treating surgeon. Ten of 11 limbs (91%) in the 3D cast group healed in an excellent position and one in an acceptable position. Eleven of 12 (92%) in the conventional cast group healed in an excellent position and one in an acceptable position. All of the fractures in the 3D cast group were found to be fully healed. At four weeks, 11 of 12 (92%) in the conventional cast group were found to be fully healed, while one was found to be partially healed radiographically.

The blinded therapist’s data is shown in Table 2. No differences were found in terms of skin irritation by the therapist. The patients’ survey responses are also shown in Table 2. Differences in skin irritation, comfort, satisfaction, and cast care favoring the 3D casts were identified (p<0.05).

The orthotists, at four weeks after application, did not find any of the orthoses (3D cast or fiberglass cast) unacceptable or in need of alteration. No treatment alteration or crossover of any patient due to complications or compliance factors was recorded. One cast in the 3D group and one in the conventional group were replaced due to a broken cast and a rash respectively.

**Table 2:** Therapist and patient forms VAS: visual analog scale

		3D cast	Conventional cast	P-value
Therapist				
Skin	Intact	9	6	0.19
	Minor irritation	2	2	
	Substantial irritation	0	4	
	Need to alter treatment	0	0	
Patient				
Skin	Intact	8	2	0.01
	Minor irritation	2	9	
	Substantial irritation	1	1	
Comfort (VAS)	0-10	9.1	7.1	0.02
Cast care	No hassle	10	2	0.0006
	Minimal burden	1	6	
	Moderate burden	0	3	
	Very difficult	0	1	
	Unacceptable	0	0	
Satisfaction (VAS)	0-10	9.4	7.2	0.009

## Discussion

While conventional casting is extremely effective, inexpensive, and practical, it has considerable limitations. Skin complications that result include itching, bacterial infections, ulceration, rashes, and contact dermatitis. Current solutions, like adding extra padding over bony prominences, may decrease these risks [7,8]. These skin complications can lead to significant discomfort for patients and may increase healthcare utilization unrelated to the initial injury [9]. Conventional casts must also stay dry and must be completely covered when bathing. However, waterproof casting, which has become more widely used, helps avoid these issues.

In addition to skin complications, routine activities of daily living and hygiene are significantly affected when a child is placed in a conventional cast. These casts tend to be bulky and uncomfortable, which can lead to frustration and dissatisfaction for pediatric patients and their parents. Children are the most affected group and are often non-compliant due to limited awareness of imposed limitations along with a lack of coordination and attention to cast care [7]. These factors contribute to pediatric cast complications.

3D-printed orthoses offer an opportunity to use digital technology to improve the patient’s experience while providing appropriate immobilization. The fit is made to match the exact anatomy of the patient by scanning the limb. This contouring avoids pressure points and allows open areas over wounds or incisions. The open areas allow ventilation to the custom-made orthosis [10]. The 3D orthosis can be fixed or removable based on the patient's needs as determined by the treating physician. We chose to use a fine layer of mesh liner under the 3D cast. This mesh liner was placed on the skin prior to the administration of the 3D cast. This allows for more breathability, decreased perspiration, and potentially less irritation from the orthosis itself. This liner is waterproof; therefore, it can stay in place at all times. It can also be removed for bathing and swimming if the patient prefers and then put back in place. We chose to include this liner to help make the two groups even more similar. Of note, in clinical practice, this liner is optional. The time for cast application should be after maximal swelling has occurred, which is typically three to five days post-injury. If a 3D orthosis is selected for treatment, it can be used for the entirety of the fracture healing episode because it allows for greater swelling and expansion through its vented windows.

In comparison, ready-made wrist splints are not contoured to the exact anatomy of each patient, which creates a risk for pressure points on the skin. Circumferential off-the-shelf braces (casts) generally do not offer the same protection and rigidity as rolled fiberglass casts [1]. In addition, ready-made wrist splints are removable and do not allow the device to be affixed for the duration of immobilization. This is not optimal for those patients who are non-compliant and require full-time cast wearing for their fracture. Most removable wrist splints are not water-resistant and can trap moisture. They do not easily allow for bathing and swimming while applied.

There are scarce data regarding the treatment of pediatric upper extremity fractures with 3D-printed casts. Guida et al. developed a protocol to manufacture customized, 3D-printed orthoses in a hospital setting [11]. Eighteen children were successfully treated with the 3D-printed cast, all with high patient satisfaction. The device was significantly lighter than a plaster cast and allowed for ventilation. The fabrication cost was comparable to a conventional plaster cast.

Graham et al. evaluated the functionality of 3D-printed orthoses compared to conventional immobilization in adults [12]. While immobilized in either a short-arm fiberglass cast or a 3D-printed orthosis, volunteers were assessed with both the Jebsen Hand Function Test (JHFT) and Patient-Rated Wrist Evaluation (PRWE). Those with the 3D orthosis were faster at completing tasks in the JHFT, and their PRWE scores were much lower, signifying less disability. Outcomes of patient satisfaction, comfort, and perceived function were superior in the 3D orthosis group. The mean wear burden was moderate in the fiberglass cast group compared with no hassle´ in the 3D cast group. Additionally, Chen et al. reported on 10 adult patients treated with a 3D-printed cast to treat distal radius fractures [3]. All of the patients opted to use the 3D-printed cast instead of a plaster cast. All patients healed after treatment in the 3D-printed cast.

There are several limitations to this study. Firstly, the number of patients in the study was small by design. This was primarily designed as a safety and proof of concept study. Secondly, we did not measure functional outcomes. Third, the follow-up was of short duration. However, patients in the age group with the types of injuries we studied fully recover in a very short period of time. Fourth, it is possible that the ‘coolness’ factor led the patients to respond more positively to the 3D-printed casts than the conventional casts. Finally, all of these fractures were non to minimally displaced and stable. The findings of the study cannot be extrapolated to more severe injuries.

## Conclusions

This pilot study was designed to evaluate the early adoption of this 3D-printing technology in this clinical setting. Based on these preliminary results, it appears that 3D-printed casts offer a safe and viable alternative to conventional casting. Further studies with a larger number of patients are required to establish the efficacy of this new technology in the treatment of traumatic injuries to the upper extremity in the pediatric population.
